# Broad-Spectrum RAS Inhibition in Pancreatic Ductal Adenocarcinoma: Mechanistic Advances and Therapeutic Promise

**DOI:** 10.3390/ph18121788

**Published:** 2025-11-24

**Authors:** Fawaz E. Alanazi, Yasser Alatawi, Abdullah Alattar, Reem Alshaman, Ahmed A. Kotb, Helal F. Hetta

**Affiliations:** 1 Department of Pharmacology and Toxicology, Faculty of Pharmacy, University of Tabuk, Tabuk 71491, Saudi Arabia; falanazi@ut.edu.sa (F.E.A.); aalattar@ut.edu.sa (A.A.); ralshaman@ut.edu.sa (R.A.); 2 Department of Pharmacy Practice, Faculty of Pharmacy, University of Tabuk, Tabuk 71491, Saudi Arabia; yasser@ut.edu.sa; 3 Department of Microbiology and Immunology, Faculty of Pharmacy, Assiut University, Assiut 71515, Egypt; ahmedabedelaziz994@aun.edu.eg; 4 Division of Microbiology, Immunology and Biotechnology, Department of Natural Products and Alternative Medicine, Faculty of Pharmacy, University of Tabuk, Tabuk 71491, Saudi Arabia

**Keywords:** Pan-Ras inhibitors, RAS(ON) inhibitors, pancreatic ductal adenocarcinoma (PDAC)

## Abstract

The RAS family of oncoproteins (KRAS, HRAS, and NRAS) drive aggressive cancers like pancreatic ductal adenocarcinoma (PDAC) and non-small cell lung cancer (NSCLC), yet targeting mutant RAS has historically been challenging due to its “undruggable” structure. Recent advances in mutation-specific inhibitors (e.g., sotorasib for KRAS^G12C^) have demonstrated clinical efficacy but face limitations in tumor types like PDAC, where KRAS^G12C^ mutations are rare. Broad-spectrum pan-RAS inhibitors (e.g., RMC-7977, RMC-6236, ADT-007/ADT-1004) now offer promise by targeting active GTP-bound or nucleotide-free RAS across isoforms and mutations. Preclinical studies show these agents induce deep tumor regressions, overcome resistance to allele-specific inhibitors, and remodel the tumor microenvironment (TME) by enhancing T-cell infiltration and reducing immunosuppressive myeloid cells. Early clinical data for RMC-6236 report disease control rates of 85–87% in NSCLC and PDAC, with manageable toxicity. This review shows that pan-RAS inhibitors represent a promising new class of therapeutics capable of overcoming many historical challenges associated with the “undruggable” nature of RAS proteins and demonstrating encouraging preclinical and early clinical results, particularly in difficult-to-treat tumor types such as PDAC and NSCLC. Challenges remain in achieving a therapeutic index due to RAS’s role in normal tissue homeostasis, but tumor-specific drug accumulation and rapid normal tissue recovery may mitigate risks. Ongoing trials are evaluating combination strategies with immunotherapy and chemotherapy, positioning pan-RAS inhibitors as transformative agents for RAS-driven cancers.

## 1. Introduction

Pancreatic ductal adenocarcinoma (PDAC) stands as the most prevalent histologic type of pancreatic cancer, accounting for 85–95% of all solid pancreatic tumors and representing a profoundly lethal malignancy [1]. Despite advances in medical technology, PDAC continues to exhibit a dismal prognosis, with a global five-year survival rate remaining below 12% for early-stage diagnoses and dropping to approximately 3% for metastatic cases [2]. The incidence and mortality rates of PDAC are steadily rising worldwide, and it is projected to become the second leading cause of cancer-related deaths in the United States by 2030, posing a growing public health burden [3,4].

Recent reviews have emphasized that PDAC is not only molecularly heterogeneous but also driven by a uniquely immunosuppressive and desmoplastic tumor microenvironment that severely limits drug penetration and immune surveillance. Fibroblast activation, extracellular matrix remodeling, and cytokine signaling sustain a pro-tumor milieu that hinders cytotoxic therapy and contributes to relapse. This complex stromal–immune interaction, characterized by hypovascularity and persistent fibroinflammatory signaling, is now recognized as a major determinant of chemoresistance and immune evasion. These biological hallmarks underline the urgent unmet medical need for molecularly targeted strategies that can overcome stromal and immune barriers in PDAC [5,6,7,8,9].

Recent epidemiological analyses have confirmed that both incidence and mortality of pancreatic cancer have more than doubled over the past three decades, with only minimal improvement in survival outcomes despite advances in multimodal therapy [7,8,9]. These trends emphasize the urgent need for novel molecularly targeted and immune-modulatory strategies capable of altering the trajectory of this disease.

A defining feature of PDAC is its marked resistance to current systemic therapies. Standard treatments, including chemotherapy agents such as gemcitabine, have yielded only marginal improvements in survival, largely due to the rapid development of both intrinsic and acquired drug resistance [10]. Multiple mechanisms underlie this resistance, including aberrant gene expression, activation or hyperactivation of key signaling pathways (such as NF-κB, HIF-1, and PI3K/Akt), epithelial–mesenchymal transition (EMT), the influence of the tumor microenvironment, and the presence of highly resistant cancer stem cells (Notch pathway) [10]. Additionally, the dense, poorly vascularized stroma of PDAC tumors creates regions of hypoxia, further promoting chemoresistance and aggressive tumor behavior [10]. These biological barriers contribute to the frequent diagnosis of PDAC at advanced stages, when curative surgical options are limited and systemic therapies are largely ineffective [1].

At the molecular level, PDAC is characterized by a high prevalence of activating mutations in the RAS proto-oncogene family, particularly KRAS, which is mutated in over 90% of cases. These mutations drive tumor progression by shifting RAS proteins toward their active, GTP-bound state, thereby activating downstream signaling pathways that promote cell proliferation and survival (PI3K/Akt and MAPK). Historically, the RAS protein family was considered undruggable, and the lack of suitable binding sites for small-molecule inhibitors has hampered efforts to target KRAS mutations in PDAC directly. While recent FDA-approved therapies targeting the KRAS^G12C^ variant have transformed the management of certain cancers, their impact on PDAC remains limited due to the low frequency (3%) of this specific mutation and the rapid emergence of adaptive resistance. Given these obstacles, novel therapeutic approaches are critically needed to overcome the complex resistance mechanisms inherent to PDAC [11,12,13].

To address these challenges, recent research has focused on the development of broad-spectrum, multiselective RAS inhibitors—commonly referred to as pan-RAS or RAS(ON) inhibitors—that target multiple RAS isoforms and mutations simultaneously. The term RAS(ON) describes inhibitors that bind to and stabilize the active, GTP-bound conformation of RAS, thereby preventing its interaction with downstream effectors such as RAF, PI3K, and RAL. In contrast, “RAS-OFF” inhibitors, such as covalent KRAS^G12C^ agents, act by locking the inactive, GDP-bound form. This mechanistic distinction allows RAS(ON) inhibitors to overcome mutation-specific limitations and maintain efficacy across diverse oncogenic RAS variants [14,15,16].

The concept of multi-target inhibition has gained increasing attention as a strategy to overcome adaptive resistance in cancer therapy. Conventional single-target kinase inhibitors often fail due to pathway redundancy and compensatory feedback loops. Multi-kinase or broad-spectrum inhibitors can counteract resistance mechanisms by simultaneously modulating multiple signaling nodes within oncogenic networks [17]. This principle of polypharmacology—targeting several molecular pathways rather than a single mutation—forms the conceptual foundation for the development of next-generation broad-spectrum RAS inhibitors and degradation-based strategies (e.g., PROTAC-mediated RAS degraders).

Recent research has focused on the development of broad-spectrum Multiselective RAS inhibitors, panRAS inhibitors, or RAS (ON) inhibitors such as RMC-7977, RMC-6236, ADT-007, and its analog ADT-1004), which offer promise by targeting active GTP-bound or nucleotide-free RAS across isoforms and mutations. These agents have demonstrated promising preclinical activity, favorable pharmacological profiles, and minimal effects on healthy tissue, positioning them as potential candidates for broader clinical application in PDAC and other RAS-driven malignancies.

By targeting both tumor-intrinsic RAS signaling and the immunosuppressive tumor microenvironment, these inhibitors represent a promising avenue for overcoming the current barriers in PDAC therapy. This review comprehensively shows that the emergence of broad-spectrum RAS(ON) inhibitors represents a promising avenue for overcoming the current obstacles in PDAC therapy.

## 2. Evolution of RAS-Targeted Therapy and Its Mechanisms

### 2.1. RAS Family and Its Role in Cancer

**Figure 1:**
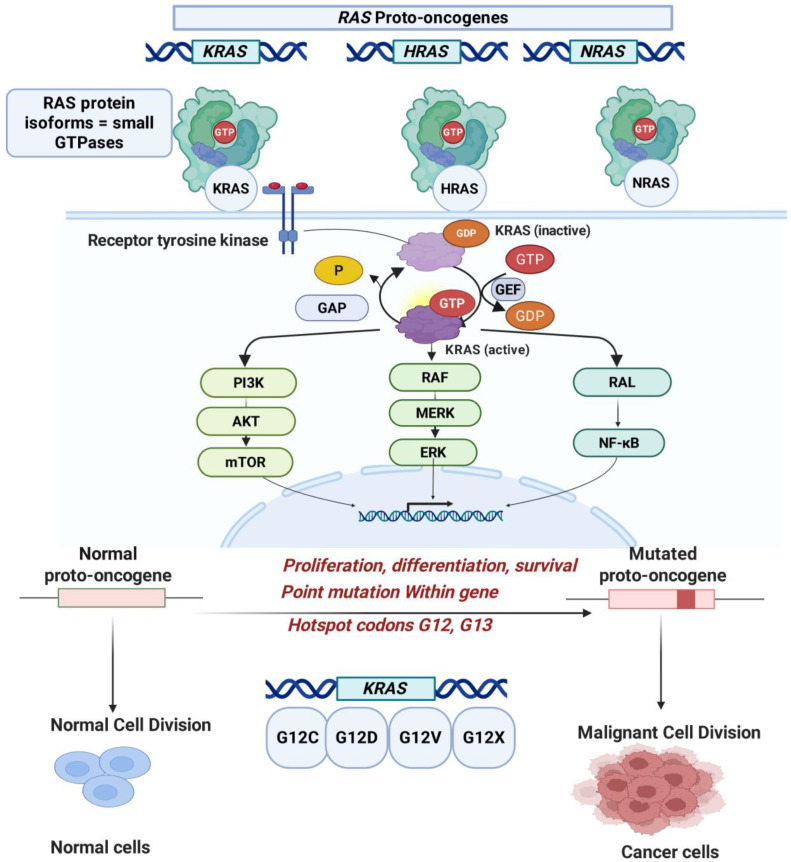
Role of RAS proto-oncogenes in cell signaling and cancer progression. RAS genes (*KRAS*, *HRAS*, and *NRAS*) encode small GTPases that regulate key cellular processes such as proliferation, differentiation, and survival. Under normal conditions, RAS proteins cycle between inactive GDP-bound and active GTP-bound states, controlled by GEFs (guanine nucleotide exchange factors) and GAPs (GTPase-activating proteins). Upon activation by receptor tyrosine kinases, RAS triggers downstream signaling pathways including PI3K/AKT/mTOR, RAF/MEK/ERK, and RAL/NF-κB. Oncogenic mutations—most commonly at codons G12 and G13—convert RAS proto-oncogenes into constitutively active oncogenes, driving uncontrolled cell division and tumorigenesis. Common KRAS mutations include G12C, G12D, G12V, and G12X, which disrupt normal signaling and promote malignant transformation. Solid arrows (→) indicate the direction of signaling or activation events; curved arrows depict cycling between inactive and active RAS states; downward arrows (↓) denote downstream biological consequences; and dashed arrows represent conceptual transitions such as progression from normal proto-oncogene to mutated oncogene (created with BioRender.com).

The RAS family of proto-oncogenes, as indicated in Figure 1, comprises three isoforms, KRAS, HRAS, and NRAS, each encoding small GTPase proteins that function as molecular switches cycling between an active GTP-bound state and an inactive GDP-bound state. This cycling regulates key signaling pathways controlling cell growth, survival, and proliferation. Activating mutations in RAS genes, particularly gain-of-function missense mutations at hotspot codons G12, G13, and Q61, shift the equilibrium toward the constitutively active RAS(ON) state, leading to increased oncogenic signaling and tumor progression. KRAS mutations are especially prevalent in pancreatic ductal adenocarcinoma (PDAC), colorectal cancer (CRC, ~40%), and non-small cell lung cancer (NSCLC, ~30%), with PDAC showing over 90% prevalence of KRAS mutations, predominantly at codon G12 (G12D, G12V, G12R). These mutations occur early in cancer development and contribute significantly to the aggressive nature of PDAC [11,12,18,19,20,21,22,23,24,25,26].

### 2.2. Challenges and Limitations of Mutation-Specific KRAS Inhibitors

Despite the critical role of KRAS mutations in cancer, targeting RAS oncoproteins has historically been difficult due to the lack of suitable binding sites on the protein surface. The recent FDA approval of two KRAS^G12C^-specific inhibitors, sotorasib and adagrasib, marked a breakthrough in targeting mutant KRAS, particularly in NSCLC. However, these inhibitors have limited applicability in PDAC because KRAS^G12C mutations account for 1–3% of cases [27,28]. Moreover, the clinical efficacy of these agents is constrained by intrinsic resistance and the development of acquired resistance [29,30,31]. Resistance mechanisms include secondary KRAS mutations, amplification of the KRAS^G12C^ allele, and activation of wild-type RAS isoforms (HRAS and NRAS) via upstream receptor tyrosine kinases (RTKs), which reactivate downstream signaling pathways [32,33,34,35].

Importantly, accumulating evidence suggests that additional oncogenic drivers and tumor suppressor gene (TSG) alterations also play crucial roles in PDAC biology and therapeutic resistance. For instance, while BRAF^V600E^ mutations are rare in human PDAC, they can initiate PanIN formation and, when combined with gain-of-function TP53 mutations, drive invasive disease in experimental models. The PI3K/Akt/mTOR pathway is another key effector downstream of KRAS; however, activating PI3K mutations alone are not strong tumorigenic drivers in PDAC and often co-occur with KRAS mutations. Furthermore, loss-of-function mutations in TSGs such as SMAD4 and TP53 frequently co-occur with KRAS mutations and are associated with accelerated tumor progression and poor prognosis. Indeed, KRAS, TP53, CDKN2A, and SMAD4 represent the only four genes mutated in more than 15% of PDAC cases, underscoring their central role in disease pathogenesis. Therefore, comprehensive molecular profiling—including assessment of BRAF^V600E^, PI3K/Akt/mTOR, STK11, and key TSGs such as SMAD4 and TP53—may help identify additional therapeutic vulnerabilities and inform more effective, personalized treatment strategies for PDAC patients [36].

### 2.3. Emergence of Pan-RAS and Broad-Spectrum Inhibitors

The limitations of mutation-specific inhibitors have shifted research focus toward broad-spectrum or pan-RAS inhibitors that target multiple RAS isoforms and mutation variants. These inhibitors aim to overcome the diverse mutational landscape and acquired resistance mechanisms observed in pancreatic ductal adenocarcinoma (PDAC) and other RAS-driven cancers. Recent advancements have led to the development of multi-selective tri-complex inhibitors or pan-RAS(ON) inhibitors such as RMC-7977 and RMC-6236 (daraxonrasib), which are currently in preclinical and early-phase clinical evaluation, respectively. These compounds specifically bind to the active GTP-bound (RAS–GTP) state of all RAS isoforms (KRAS, HRAS, and NRAS), encompassing both mutant and wild-type forms [37,38,39,40], as summarized in Table 1.

Mechanistically, these agents form a tri-complex with cyclophilin A (CypA) and RAS–GTP, locking RAS in a conformationally restricted yet GTP-bound state that prevents effector interaction. By stabilizing the active RAS(ON) form in a signaling-incompetent configuration, tri-complex inhibitors such as RMC-6236 and RMC-7977 block effector engagement with PI3K (phosphoinositide 3-kinase), RAF kinases, and RAL-GDS (RAL guanine nucleotide dissociation stimulator), thereby silencing downstream MAPK and PI3K/AKT signaling. This approach differs fundamentally from mutation-specific covalent inhibitors (e.g., KRAS^G12C^) that target the GDP-bound RAS(OFF) state; instead, tri-complex inhibitors neutralize the oncogenic signal by preventing RAS(ON) from communicating with its effectors. Structurally, the inhibitor occupies the switch II pocket (SII-P) of RAS and recruits CypA to create a nonproductive ternary complex, stabilizing RAS–GTP and reducing its dynamic flexibility necessary for effector binding [37,38,39,40].

**Figure 2:**
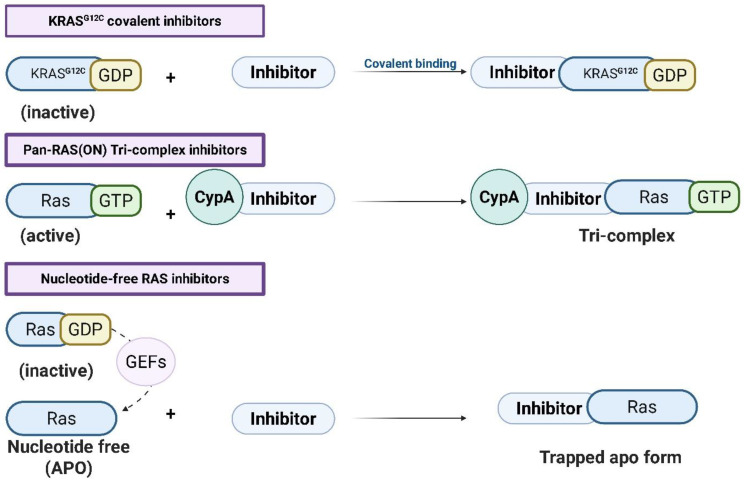
Mechanisms of RAS-targeting inhibitors. Three classes of RAS inhibitors are depicted based on their target conformations and mechanisms of action: (1) KRAS^G12C^ covalent inhibitors bind irreversibly to the inactive GDP-bound KRAS^G12C^ mutant via covalent interaction. (2) Pan-RAS(ON) tri-complex inhibitors stabilize the active GTP-bound RAS conformation by forming a ternary complex with cyclophilin A (CypA) and the inhibitor. (3) Nucleotide-free RAS inhibitors trap RAS in the nucleotide-free (apoRAS or nfRAS) state, preventing reactivation by guanine nucleotide exchange factors (GEFs) and stabilizing an inactive form. Straight arrows (→) indicate the direction of the binding or complex-formation process, while dashed arrows represent GEF-mediated nucleotide exchange leading to the apo state (created with BioRender.com).

This strategy, distinct from covalent KRAS^G12C^ inhibition, neutralizes RAS signaling at its active state rather than blocking activation, resulting in durable pathway inhibition and broad isoform coverage, as illustrated in Figure 2 [37,38,39,40].

### 2.4. Nucleotide-Free State Targeting: ADT-007 and ADT-1004

A complementary approach involves targeting the nucleotide-free (apoRAS) form of RAS proteins, as also illustrated in Figure 2. During the nucleotide exchange cycle, RAS transiently adopts an apo state after GDP release and before GTP binding. This conformation is normally short-lived and catalyzed by guanine nucleotide exchange factors (GEFs) such as SOS1. Compounds like ADT-007 and its orally bioavailable prodrug ADT-1004 exploit this vulnerable intermediate by binding to apoRAS and preventing GTP loading, effectively trapping RAS in an inactive, nucleotide-free conformation. This “RAS-off trapping” locks the protein outside the signaling cycle, blocking the reformation of RAS–GTP and shutting down downstream MAPK (mitogen-activated protein kinase) and AKT (protein kinase B) cascades. Biochemically, these inhibitors occupy a hydrophobic pocket adjacent to the P-loop and switch regions, displacing residues necessary for nucleotide binding, as demonstrated in recent biochemical and structural studies. By targeting a universal transient intermediate common to all RAS isoforms, apoRAS inhibitors achieve mutation-agnostic inhibition and may suppress RAS-driven signaling more completely than allele-specific agents [41,42,43,44,45].

**Table 1:** Summary of key pan-RAS–targeting compounds, their developers, and mechanisms of action.

Compound	Developer/Sponsor	Mechanism/Target State	Refs.
RMC-6236 (daraxonrasib)	Revolution Medicines Inc., Redwood City, CA, USA	Tri-complex RAS(ON) inhibitor—engages active, GTP-bound RAS to block effector (RAF/PI3K/RAL) interactions across multiple KRAS mutations.	[14,16,40,46,47]
RMC-7977	Revolution Medicines Inc., Redwood City, CA, USA	Tri-complex pan-RAS(ON) inhibitor—preclinical tool compound that stabilizes RAS(GTP)–chaperone complex and prevents downstream signaling.	[14,16,40]
ADT-007	ADT Pharmaceuticals, LLC, Orange Beach, AL, USA	Nucleotide-free (apo-RAS) inhibitor—traps nucleotide-free RAS, blocking GDP/GTP re-loading and effector activation; broad isoform selectivity.	[41,48]
ADT-1004	ADT Pharmaceuticals, LLC, Orange Beach, AL, USA (oral prodrug of ADT-007)	Nucleotide-free (prodrug) inhibitor—improves solubility and pharmacokinetics; releases ADT-007 in vivo to inhibit apo-RAS and remodel the tumor microenvironment (TME) in PDAC models.	[41]

In preclinical models, ADT-007 induced apoptosis and G2/M cell-cycle arrest in PDAC and colorectal cancer cell lines, with broad suppression of phospho-cRAF, phospho-MEK, phospho-ERK, and phospho-AKT—consistent with pan-pathway inhibition. Its prodrug, ADT-1004, exhibited improved pharmacokinetic stability and potent tumor regression in PDAC xenografts and syngeneic models. Notably, ADT-1004 also remodeled the tumor microenvironment (TME), enhancing dendritic cell activation, shifting macrophage polarization toward an M1-like phenotype, and promoting cytotoxic T-cell infiltration. These immunomodulatory effects suggest that combining apoRAS inhibitors with immune checkpoint blockade (e.g., anti-PD-1, anti-CTLA-4, anti-LAG-3) could achieve synergistic tumor control [41,42].

### 2.5. Emerging RAS Degradation Strategies

To complement the strategy of targeting mutant RAS through small-molecule inhibitors, the emergence of RAS degraders has become a rapidly advancing and innovative therapeutic concept. Given the central role of RAS in regulating cell proliferation and survival, the complete removal of the RAS protein represents a promising means of suppressing RAS-driven oncogenic signaling. A variety of degrader molecules are currently being developed, including those that target all RAS isoforms simultaneously (pan-RAS degraders) as well as agents selective for specific isoforms or mutant variants such as KRAS. Early-stage research and preclinical evaluations, and in some cases ongoing clinical trials, have explored multiple classes of RAS degraders, notably PROTACs, linker-based systems, and direct proteolysis approaches [49].

PROTACs (proteolysis-targeting chimeras) constitute a novel class of bifunctional small molecules that induce degradation rather than inhibition of a protein of interest. Each PROTAC molecule possesses one ligand that binds the target protein and another that engages an E3 ubiquitin ligase complex. This dual engagement brings the target protein—such as RAS—into proximity with an E3 ligase substrate receptor, commonly cereblon (CRBN), facilitating ubiquitination and subsequent proteasomal degradation. This mechanism provides a mechanistically distinct and highly selective route to eliminate oncogenic RAS proteins [49].

The targeted protein degradation (TPD) strategy offers several theoretical advantages over conventional inhibition. Instead of transiently blocking enzymatic or signaling activity, degraders physically remove the oncoprotein from the cell, potentially preventing adaptive resistance that often arises through compensatory pathway reactivation. Moreover, pan-RAS degraders have the potential to achieve broad suppression of RAS signaling across KRAS, NRAS, and HRAS isoforms, paralleling the multitarget rationale of broad-spectrum kinase or RAS(ON) inhibitors. Collectively, these advances establish RAS degraders—especially PROTAC-based architectures—as a promising next-generation approach for durable, mutation-agnostic blockade of oncogenic RAS signaling [49].

### 2.6. Mechanistic Comparison of RAS-Targeted Therapies

The differential sensitivity of KRAS^G12X^ mutants to various RAS inhibitors can be attributed to several key mechanistic factors. First, codon 12 mutant RAS proteins demonstrate higher affinity for the GTP-bound state, which can either enhance or reduce their activity and thus influences their interaction with inhibitors targeting the active conformation of RAS. This molecular characteristic makes them particularly susceptible to inhibitors designed to bind RAS in its active conformation [19].

Second, the biochemical properties of specific KRAS mutations vary significantly, affecting both their intrinsic GTPase activity and their interactions with regulatory proteins. These distinct biochemical profiles contribute to differences in inhibitor efficacy across various KRAS mutation subtypes [50].

Third, the overall RAS dependency of cancer cells plays a crucial role in determining inhibitor sensitivity. This dependency is not uniform across all RAS-mutant cancers but is heavily influenced by tissue-specific phenotypes and co-mutation status. The genetic context in which KRAS mutations occur can significantly alter signaling networks and cellular responses to RAS inhibition [51,52].

NSCLC and PDAC harboring KRAS^G12C^ mutations have been shown to be particularly dependent on oncogenic RAS signaling. This dependency is evidenced by both preclinical studies investigating KRAS^G12D^ inhibition and inactivation and by the clinical success of KRAS^G12C^ inhibitors in treating KRAS^G12C^-mutated NSCLC. The FDA approval of sotorasib and adagrasib as monotherapies for KRAS^G12C^-mutated NSCLC underscores the critical role of RAS signaling in these specific cancer contexts [29,31,53,54].

A recent study has demonstrated that co-mutations in STK11, KRAS, and TP53 significantly influence prognosis and therapeutic outcomes in NSCLC. Specifically, when STK11 is co-mutated with KRAS, patients experience notably worse progression-free and overall survival than those with STK11 mutation alone, highlighting a deleterious interaction between these genetic alterations. In contrast, the presence of TP53 co-mutation with STK11—even in the context of KRAS mutation—confers a better prognosis, suggesting that the interplay between these tumor suppressors and oncogenes is complex and context-dependent [55].

These findings underscore the importance of comprehensive molecular profiling to predict patient outcomes better and guide therapeutic decision-making in NSCLC. Moreover, such mechanistic insights highlight the necessity of considering both the specific RAS mutation and the broader cellular context when developing and applying RAS-targeted therapies, which helps to explain why certain inhibitors may demonstrate greater efficacy in specific cancer types or against particular KRAS mutations.

## 3. Preclinical and Clinical Insights on Pan-RAS Inhibitors

### 3.1. Promising Activity of RMC-7977, RMC-6236, and ADT-007/ADT-1004 in KRAS-Mutated Cancers

Recent preclinical and clinical investigations have revealed the promising effectiveness of pan-RAS inhibitors, specifically RMC-7977 and RMC-6236, in targeting KRAS^G12X^-mutated malignancies, including non-small cell lung cancer (NSCLC) and pancreatic ductal adenocarcinoma (PDAC), as summarized in Table 2.

**Table 2:** Overview of major preclinical and clinical studies of pan-RAS inhibitors, highlighting study phase, tumor models, and key efficacy and safety outcomes.

Compound	Study Type/Model	Trial Identifier & Title	Phase/Status (as of 2025)	Population/Design	Main Outcomes (Efficacy)	Safety/Notable AEs	Ref.
RMC-6236 (daraxonrasib)	Clinical, solid tumors incl. PDAC & NSCLC	NCT05379985—A Study of RMC-6236 in Subjects with Advanced Solid Tumors Harboring KRAS^G12X^ Mutations	Phase I—Active, recruiting (2025)	Open-label dose-escalation/expansion; monotherapy and combination arms	Interim data: NSCLC ORR ≈ 38%, DCR ≈ 85%; PDAC ORR ≈ 20%, DCR ≈ 87%	Mostly G1-2 rash, diarrhea, nausea; rare G3-4 events (<5%)	[46,47]
	Planned confirmatory	NCT06625320—RMC-6236 vs. Physician’s Choice in Metastatic PDAC After First-Line Therapy	Phase III—Recruiting (2025)	Randomized (2 arms); primary endpoint PFS	Ongoing—no results yet	—	
RMC-7977	Preclinical models (KRAS-mutant PDAC & NSCLC)	—	Preclinical—tool compound	Murine xenograft & cell-line studies	Tumor growth inhibition & pathway suppression (pERK↓ pAKT↓)	Well tolerated in preclinical PK/Tox assays	[14,16]
ADT-007	Preclinical (PDAC and CRC cell lines; xenografts)	—	Preclinical	Compared with KRAS G12C inhibitors in vitro and in vivo	Potent RAS-GTP suppression; induced apoptosis and G2/M arrest; tumor regression in PDAC PDX models	Limited solubility and rapid clearance prompted the ADT-1004 design	[41,48]
ADT-1004 (oral prodrug of ADT-007)	Preclinical (PDAC orthotopic and PDX models)	—	Preclinical	Evaluated alone and with anti-PD-1 therapy	Enhanced tumor regression and TME reprogramming (CD4^+^/CD8^+^ T-cell infiltration, M2→M1 shift)	Improved PK; favorable tolerability	[41,48]

(↓) indiate downregulation/inhibition.

RMC-7977, a multi-selective inhibitor targeting the active GTP-bound state of all RAS isoforms, has shown greater antitumor activity compared to agents targeting upstream (SHP2) or downstream signaling proteins, such as MEK1/2 and ERK1/2. This enhanced efficacy is attributed to the direct targeting of the RAS oncoprotein itself, enabling more efficient suppression of oncogenic RAS signaling. RMC-7977 effectively inhibits tumor growth in KRAS^G12X^-mutant cancers by exploiting the pronounced oncogene addiction characteristic of RAS-driven tumors [14].

Data from pilot clinical trials of RMC-6236, a structurally analogous clinical drug, further corroborate the therapeutic potential of pan-RAS inhibitors. In patients with NSCLC, RMC-6236 attained a disease control rate (DCR) of 85% and an objective response rate (ORR) of 38%. In patients with PDAC, the Disease Control Rate (DCR) and Objective Response Rate (ORR) were 87% and 20%, respectively [47]. These results highlight meaningful clinical activity in two of the most challenging RAS-driven cancers.

Concerning safety, grade 4 treatment-related adverse events were infrequent, with one instance of large intestine perforation occurring at the site of invasive PDAC. Discontinuation of treatment due to adverse effects was observed in 5% of patients, with grade 3 rash, nausea, diarrhea, and vomiting identified as the predominant treatment-related problems [47].

Oral administration of ADT-1004 demonstrated significant antitumor activity across multiple PDAC models, comprising orthotopically implanted murine cell lines and patient-derived xenografts (PDXs) possessing various KRAS mutations. The therapy successfully suppressed RAS activation and subsequent MAPK signaling, as seen by reduced RAS-GTP and pERK levels. ADT-007 induced apoptosis and G2/M cell-cycle arrest in pancreatic ductal adenocarcinoma (PDAC) and colorectal cancer cell lines, while concurrently suppressing the phosphorylation of key RAS pathway effectors—cRAF (cellular rapidly accelerated fibrosarcoma kinase), MEK (mitogen-activated protein kinase kinase), AKT (protein kinase B), and ERK (extracellular signal-regulated kinase)—demonstrating broad inhibition of downstream RAS signaling [48]. These findings align with the demonstrated efficacy of various mutation-specific and pan-RAS inhibitors in pancreatic ductal adenocarcinoma (PDAC) [46,54,56].

ADT-007 is a reversible, highly powerful, and selective pan-RAS inhibitor that addresses the limitations of mutation-specific inhibitors by targeting the nucleotide-free (apoenzyme) state of RAS, therefore inhibiting GTP loading and activation. To address the poor water solubility and quick metabolism of ADT-007, ADT-1004 was formulated as an orally accessible prodrug, attaining sustained plasma concentrations appropriate for once or twice daily administration in preclinical mice [41].

ADT-007 and ADT-1004 exhibited significant cytotoxicity against PDAC cell lines that are resistant to FDA-approved allele-specific KRAS inhibitors, including sotorasib and adagrasib (KRAS^G12C^ inhibitors) as well as MRTX1133 (KRAS^G12D inhibitor). Resistance to these treatments frequently emerges from secondary KRAS mutations, activation of wild-type KRAS alleles, or compensatory signaling through co-expressed RAS isoforms (NRAS, HRAS) stimulated by growth factors prevalent in the tumor microenvironment (TME). Pan-RAS inhibitors circumvent these resistance mechanisms by widely targeting all RAS isoforms [41,48].

ADT-007 entirely suppressed colony formation in KRAS^G12C^ PDAC cells, surpassing the efficacy of sotorasib, adagrasib, the KRAS-specific inhibitor BI-2865, and the pan-RAS inhibitor RMC-6236. In various PDAC models containing KRAS^G12D^ and KRAS^G12C^ mutations, ADT-007 demonstrated greater growth inhibitory efficacy than RMC-6236, indicating that targeting the nucleotide-free RAS state may yield improved anticancer effectiveness compared to inhibitors that interact with RAS in either its active or inactive forms [41,48].

### 3.2. Impact on the Tumor Microenvironment (TME)

**Figure 3:**
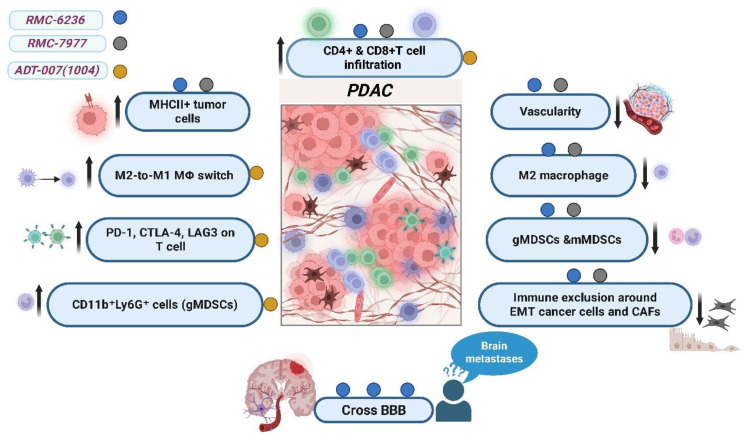
Effects of pan-RAS inhibitors RMC-6236, RMC-7977, and ADT-007 (1004) on the tumor microenvironment in pancreatic ductal adenocarcinoma (PDAC). This illustration summarizes the impact of each RAS-targeted agent on innate immune cells, adaptive immune cells, and tumor cells within the PDAC microenvironment. All three agents enhance infiltration of CD4-positive and CD8-positive T cells and increase the expression of major histocompatibility complex class II (MHC II) molecules on tumor cells, indicating improved antigen presentation. ADT-007 (1004) specifically promotes a phenotypic switch of tumor-associated macrophages from the immunosuppressive M2-like state to a pro-inflammatory M1-like state. It also increases the expression of immune checkpoint receptors—programmed cell death protein 1 (PD-1), cytotoxic T-lymphocyte-associated protein 4 (CTLA-4), and lymphocyte-activation gene 3 (LAG-3)—on T cells, reflecting immune activation. Moreover, it enhances the presence of granulocytic myeloid-derived suppressor cells (CD11b-positive Ly6G-positive cells), which may act as antigen-presenting cells. Both RMC-6236 and RMC-7977 reduce tumor vascularity, decrease levels of M2-like macrophages and monocytic and granulocytic myeloid-derived suppressor cells, and alleviate immune cell exclusion around epithelial-to-mesenchymal-transitioned cancer cells and cancer-associated fibroblasts. Notably, RMC-6236 crosses the blood–brain barrier, suggesting therapeutic potential for patients with brain metastases harboring RAS mutations. downward arrows (↓) indicate downregulation or decrease, upward arrows (↑) indicate upregulation or increase (created with BioRender.com).

Pan-RAS(ON) inhibitors demonstrate favorable pharmacokinetic and pharmacodynamic properties, including successful delivery and accumulation within tumor tissues, overcoming the biophysical barriers imposed by the TME. Treatment with these agents induces a prominent desmoplastic response and significantly reduces tumor vascularity. Notably, pan-RAS inhibition alters the immunological composition of the tumor microenvironment, as illustrated in Figure 3, by enhancing the infiltration of CD4^+^ and CD8^+^ T cells and raising the quantity of MHC class II-positive tumor cells. Simultaneously, there is a decrease in immunosuppressive cells, such as monocytic and granulocytic myeloid-derived suppressor cells and M2 macrophages, which may augment anticancer immunity [46,56,57]. Markedly, RMC-6236 has demonstrated the ability to cross the blood–brain barrier, with concentrations increasing in a dose-dependent manner. This property opens the possibility of treating patients with brain metastases harboring RAS mutations [46].

Oncogenic KRAS promotes an immunosuppressive tumor microenvironment (TME) in PDAC, whereas inhibition of RAS signaling can partially reverse this phenotype. Treatment with ADT-1004 enhanced the infiltration of both CD4^+^ and CD8^+^ T cells into the TME and simultaneously increased the expression of immune checkpoint receptors—PD-1, CTLA-4, and LAG-3—on these lymphocytes. This pattern indicates heightened immune activation, while also suggesting the emergence of compensatory inhibitory feedback mechanisms. These findings parallel those observed with mutant-selective KRAS inhibitors, such as MRTX1133, in which PD-1 upregulation renders tumors more susceptible to immune checkpoint blockade [58,59,60].

Furthermore, ADT-1004 reprogrammed macrophage populations from an immunosuppressive M2-like phenotype to a proinflammatory M1-like state and enhanced dendritic cell (DC) subsets critical for effective antigen presentation. The population of granulocytic CD11b^+^ (cluster of differentiation 11b-positive) Ly6G^+^ (lymphocyte antigen 6 complex locus G-positive) cells also increased, displaying moderate expression of MHC II (major histocompatibility complex class II) and reduced levels of PD-L1 (programmed death-ligand 1). These alterations indicate enhanced antigen-presenting capacity, which has been associated with improved survival in several cancer types [61,62]. Spatial profiling further revealed that ADT-1004 modified immune-cell localization within tumors, notably reducing immune exclusion around mesenchymal-transitioned cancer cells and cancer-associated fibroblasts (CAFs). This spatial reorganization may facilitate improved immune-mediated tumor control [41,48].

## 4. Mechanisms of Resistance to Broad RAS(ON) Inhibitors

When RAS signaling is inhibited, cancer cells can escape dependency on KRAS through compensatory mechanisms involving activation of the transcriptional coactivators YAP (Yes-associated protein) and TAZ (transcriptional coactivator with PDZ-binding motif). Activation of the YAP/TAZ–TEAD (TEA domain transcription factor) complex promotes survival and proliferation by suppressing proapoptotic genes such as PUMA, BCL2L11, and BMF, while maintaining downstream PI3K/AKT/mTOR signaling. This adaptive response has been reported across several targeted agents, including mutation-specific KRAS^G12C^, EGFR, MEK, and SHP2 inhibitors, where YAP/TAZ activation compensates for RAS blockade. In the context of broad pan-RAS(ON) inhibitors such as RMC-6236 or RMC-7977, activation of YAP/TAZ may still emerge as a bypass pathway; however, because these agents suppress multiple RAS isoforms (KRAS, NRAS, and HRAS) and conformational states simultaneously, opportunities for classical pathway reactivation are substantially reduced. Therefore, inhibition of the YAP/TAZ–TEAD axis in combination with pan-RAS inhibition has been proposed as a rational approach to prevent or delay resistance. In addition to intrinsic signaling adaptations, alterations within the tumor microenvironment (TME)—including upregulation of immunosuppressive mediators such as IL-6 and PD-L1 (programmed death-ligand 1) or expansion of myeloid-derived suppressor cells (MDSCs)—can further promote immune evasion and therapeutic resistance, underscoring the multifactorial nature of resistance mechanisms in KRAS-mutant cancers [63,64,65,66].

Unlike mutation-specific RAS inhibitors, which frequently encounter resistance through secondary mutations or reactivation of the same RAS–MAPK signaling cascade, broad-spectrum RAS(ON) inhibitors such as RMC-7977 tend to drive a more limited and predictable evolutionary escape route. Preclinical analyses of tumors that relapsed after initial responses to RMC-7977 revealed that resistant clones displayed focal amplification of the MYC oncogene. This transcriptional driver supports cell proliferation even under upstream RAS inhibition. Transcriptomic profiling also demonstrated enrichment of genes responsive to the YAP/TAZ–TEAD complex, indicating reactivation of this pathway as a key compensatory mechanism under sustained RAS suppression. Additional resistance features included epithelial–mesenchymal transition (EMT) and dedifferentiation, marked by loss of epithelial markers and decreased expression of thyroid transcription factor 1 (TTF-1), reflecting a shift toward a more invasive, stem-like phenotype. Moreover, receptor tyrosine kinase (RTK) hyperactivation, particularly through an autocrine hepatocyte growth factor (HGF)–MET (mesenchymal–epithelial transition factor) feedback loop, was identified as another driver of pathway reactivation and survival signaling [16].

Collectively, these findings indicate that while pan-RAS(ON) inhibitors cannot entirely prevent adaptive escape, they impose a more constrained evolutionary landscape for tumor resistance compared with mutation-specific RAS inhibitors. By targeting multiple RAS isoforms and activation states, these agents restrict the diversity of potential escape mutations and signaling rewiring events, resulting in a more stable and durable suppression of oncogenic RAS signaling. This mechanistic advantage positions broad-spectrum RAS(ON) inhibitors as promising therapeutic options with the potential to overcome or delay the emergence of drug resistance in RAS-driven cancers.

## 5. Combination Therapy Potential

**Figure 4:**
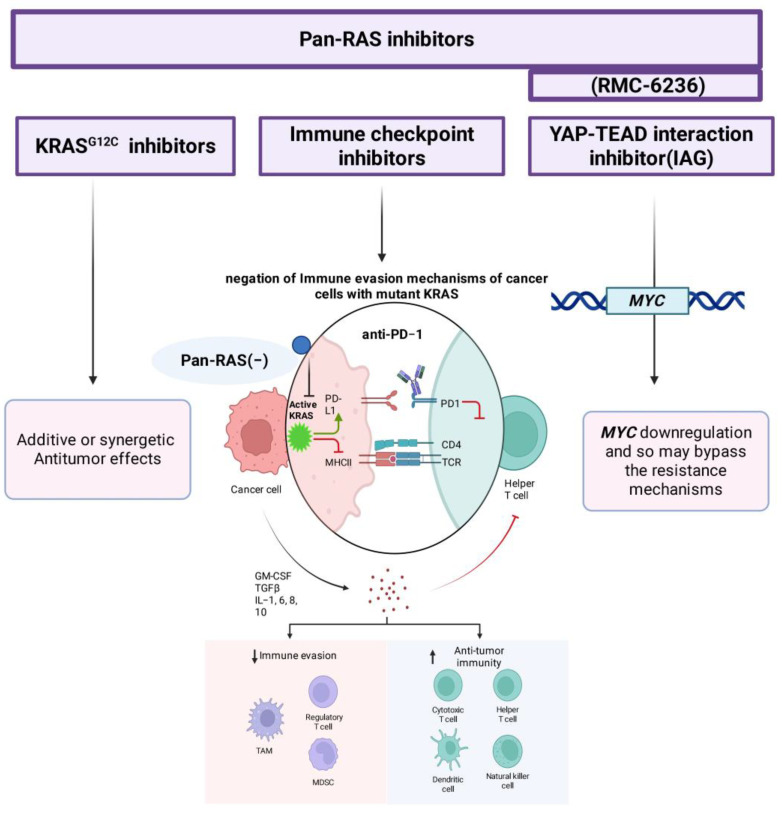
Potential combination strategies targeting RAS-driven immune evasion in cancer. Pan-RAS inhibitors (e.g., RMC-6236) exhibit enhanced antitumor effects when used in combination with mutant-selective KRAS^G12C^ inhibitors, immune checkpoint inhibitors (e.g., anti-PD-1), or YAP–TEAD interaction inhibitors (e.g., IAG933). In RAS-mutant tumors, pan-RAS inhibition suppresses oncogenic KRAS signaling, restoring MHC-I expression and reducing PD-L1 levels on tumor cells. This sensitizes them to immune checkpoint blockade, thereby negating immune evasion mechanisms and promoting T-cell-mediated antitumor responses. Simultaneous inhibition of YAP–TEAD can downregulate MYC, potentially overcoming resistance and improving therapeutic durability Blunt-ended arrows (⊣) indicate inhibitory interactions, downward arrows (↓) indicate downregulation, upward arrows (↑) indicate upregulation, and straight arrows (→) indicate directional signaling or relationships (created with BioRender.com).

Early evidence suggests that combining pan-RAS inhibitors with mutant-selective inhibitors or immune checkpoint blockade may yield additive or synergistic antitumor effects, as shown in Figure 4. Counteracting immune-evasive mechanisms driven by oncogenic KRAS sensitizes tumors to immunotherapy, supporting the rationale for combination regimens in NSCLC and other RAS-driven cancers [46,57,67,68].

Another translational challenge is maximizing efficacy while minimizing resistance and toxicity. Preclinical and early clinical evidence suggests that combining pan-RAS inhibitors with other agents-especially immunotherapies, may yield superior oncological outcomes compared to monotherapy [57]. This synergy is thought to result from the ability of RAS inhibition to negate the immune-evasive effects of oncogenic KRAS, thereby sensitizing tumors to immune checkpoint blockade [67,68]. Ongoing studies are evaluating RMC-6236 in combination regimens across various RAS-mutant solid tumors, with early results showing promise [46].

In vitro experiments combining RMC-7977 with the YAP–TEAD interaction inhibitor IAG933 demonstrated suppression of MYC expression, highlighting a promising combination therapy approach to bypass resistance mechanisms. This suggests that targeting both RAS signaling and YAP-TEAD-mediated transcription may improve therapeutic durability [46,56,57].

## 6. Clinical Translational Challenges

A central challenge in the clinical translation of broad RAS(ON) inhibitors is the essential role of RAS signaling in normal tissue homeostasis, raising concerns about achieving a suitable therapeutic index [69,70]. Despite this, preclinical and early clinical data indicate that these agents are generally well tolerated. Several factors contribute to this favorable tolerability profile: (1) The drugs tend to accumulate specifically within tumor tissue, minimizing systemic exposure [14,46,56]; (2) they demonstrate limited effects on healthy tissues [46,56], likely due to (a) lower RAS-GTP levels in normal cells, and (b) decreased affinity of broad-spectrum inhibitors for wild-type RAS in comparison to mutant versions [14,71]; (c) the ability of normal tissues to rapidly restore homeostatic equilibrium after transient RAS pathway inhibition [46,72,73].

Early clinical experience with RMC-6236 corroborates these findings: merely 1% of patients encountered a grade 4 treatment-related adverse event (large intestine perforation at the site of invasive PDAC), while 5% discontinued treatment due to adverse effects, predominantly grade 3 rash, nausea, diarrhea, and vomiting. In general, these side effects were controllable, and most patients tolerated the therapy effectively [47].

However, the potential for broad RAS inhibition to disrupt normal tissue homeostasis remains a significant concern. The challenge lies in ensuring that these agents can be used with an acceptable safety margin, especially as dosing and combination strategies are optimized in ongoing trials [41,48,74].

At present, six open-label multicenter clinical trials are in progress to evaluate the tolerability, dosage, and efficacy of RMC-6236, comprising three phase Ib trials, two phase I/II trials, and one phase III trial. Four of these studies are focused on combination therapies, reflecting the growing recognition of the need for multi-agent approaches in RAS-driven cancers. One phase III trial (NCT06625320) is evaluating RMC-6236 versus standard chemotherapy in metastatic PDAC, a setting where adaptive feedback via EGFR and other mechanisms often limits the efficacy of RAS inhibition alone [75,76].

The outcomes of these trials will be essential in determining the therapeutic efficacy and safety of RAS(ON) multi-selective tri-complex inhibitors. These findings are anticipated to establish the groundwork for incorporating pan-RAS inhibitors into conventional oncology treatment, especially in tumors like PDAC and NSCLC, where RAS mutations propel aggressive illness and existing therapeutic alternatives are scarce [41,48,74].

## 7. Conclusions

The development of pan-RAS inhibitors marks a paradigm shift in targeting oncogenic RAS, addressing the limitations of mutation-specific agents. RMC-6236, RMC-7977, and ADT-1004/ADT-007 demonstrate potent antitumor activity across diverse KRAS mutations, including those resistant to FDA-approved inhibitors. Their ability to remodel the TME and synergize with immunotherapy highlights dual mechanisms of action: direct oncogene suppression and immune activation. While tolerability challenges persist due to RAS’s role in homeostasis, early clinical data suggest a manageable safety profile, supported by tumor-specific drug accumulation and reduced affinity for wild-type RAS. These advances underscore the potential of pan-RAS inhibition to overcome adaptive resistance and expand therapeutic options for historically recalcitrant cancers like PDAC.

Nevertheless, current evidence remains preliminary, and several limitations must be acknowledged. Most available data are derived from small, early-phase clinical cohorts lacking randomized comparisons, which limits the strength of efficacy and safety conclusions. The long-term effects of broad RAS inhibition on normal tissue homeostasis and potential on-target toxicities remain incompletely understood, particularly with chronic administration or combination regimens. Moreover, the absence of validated predictive biomarkers poses a major challenge for patient selection and response monitoring. Future large-scale randomized trials integrating translational endpoints and biomarker analyses will be essential to fully define the therapeutic window, durability of response, and optimal clinical positioning of pan-RAS inhibitors.

## 8. Future Perspectives

- Clinical Trial Optimization:

Ongoing phase I–III trials of RMC-6236 must clarify dosing, safety, and efficacy in metastatic settings, particularly in PDAC and CRC, where adaptive feedback via EGFR and other RTKs complicates treatment.

- Combination Strategies:

Synergy with immune checkpoint inhibitors (e.g., anti-PD-1) and targeted agents (e.g., YAP–TEAD inhibitors) warrants exploration to counteract resistance mechanisms such as MYC amplification and EMT.

- Biomarker Development:

Identifying predictive biomarkers (e.g., MHC-II expression, T-cell clonality) will be critical for selecting patients most likely to benefit from pan-RAS inhibitors.

- TME Modulation:

Further research into how RAS inhibition alters immune cell spatial distribution and stromal interactions could inform rational combinations with stromal-targeting agents.

- Next-Generation Inhibitors:

Beyond small-molecule inhibitors, novel RAS-targeting modalities such as PROTAC-based degraders and molecular glue degraders represent a promising frontier. These approaches promote selective degradation of mutant and wild-type RAS proteins through ubiquitin–proteasome-mediated pathways, effectively silencing oncogenic signaling. Emerging pan-RAS degraders have demonstrated the potential to target multiple isoforms (KRAS, NRAS, and HRAS), providing a broader therapeutic window across diverse RAS-driven malignancies. Continued optimization of degrader selectivity, cellular permeability, and pharmacokinetic stability will be essential for clinical translation.

- Neoadjuvant Applications:

Testing pan-RAS inhibitors and degraders in early-stage disease to target micro-metastases and reduce recurrence risk could redefine curative-intent strategies.

By addressing these priorities, pan-RAS inhibitors and degraders could transition from investigation agents to cornerstone therapies, transforming outcomes for patients with RAS-mutant cancers.

## Data Availability

Not applicable.

## Abbreviations

ADT	Compound prefix used by ADT Therapeutics LLC (developer of ADT-007 and ADT-1004)
AKT	Protein Kinase B
AML	Acute Myeloid Leukemia
BBB	Blood–Brain Barrier
CAF	Cancer-Associated Fibroblast
CD	Cluster of Differentiation
CDK	Cyclin-Dependent Kinase
CRC	Colorectal Cancer
CRBN	Cereblon
CTLA-4	Cytotoxic T-Lymphocyte-Associated Protein 4
CypA	Cyclophilin A
DC	Dendritic Cell
DCR	Disease Control Rate
EGFR	Epidermal Growth Factor Receptor
EMT	Epithelial–Mesenchymal Transition
ERK	Extracellular Signal-Regulated Kinase
FDA	Food and Drug Administration
GAP	GTPase-Activating Protein
GDP	Guanosine Diphosphate
GEF	Guanine Nucleotide Exchange Factor
GTP	Guanosine Triphosphate
HGF	Hepatocyte Growth Factor
HRAS	Harvey Rat Sarcoma Viral Oncogene Homolog
IL-6	Interleukin-6
KRAS	Kirsten Rat Sarcoma Viral Oncogene Homolog
LAG-3	Lymphocyte Activation Gene 3
MAPK	Mitogen-Activated Protein Kinase
MDSC	Myeloid-Derived Suppressor Cell
MEK	Mitogen-Activated Protein Kinase Kinase
MHC	Major Histocompatibility Complex
mTOR	Mechanistic Target of Rapamycin
MYC	Myelocytomatosis Viral Oncogene Homolog
NF-κB	Nuclear Factor Kappa B
NK	Natural Killer (cells)
NRAS	Neuroblastoma Rat Sarcoma Viral Oncogene Homolog
NSCLC	Non–Small Cell Lung Cancer
ORR	Objective Response Rate
PD-1	Programmed Cell Death Protein 1
PDAC	Pancreatic Ductal Adenocarcinoma
PD-L1	Programmed Death-Ligand 1
PDX	Patient-Derived Xenograft
PI3K	Phosphoinositide 3-Kinase
PK	Pharmacokinetics
PROTAC	Proteolysis-Targeting Chimera
RAF	Rapidly Accelerated Fibrosarcoma Kinase
RAL-GDS	Ral Guanine Nucleotide Dissociation Stimulator
RAS	Rat Sarcoma Viral Oncogene Family
RTK	Receptor Tyrosine Kinase
SII-P	Switch II Pocket
SHP2	Src Homology Region 2–Containing Protein Tyrosine Phosphatase 2
SOS1	Son of Sevenless Homolog 1
STK11	Serine/Threonine Kinase 11
TAZ	Transcriptional Co-Activator with PDZ-Binding Motif
TEAD	TEA Domain Transcription Factor
TGF	Transforming Growth Factor
TME	Tumor Microenvironment
TP53	Tumor Protein p53
TSG	Tumor Suppressor Gene
YAP	Yes-Associated Protein
